# Recommendations from guidelines for the prevention of venous thromboembolism in pregnant women with inherited thrombophilia

**DOI:** 10.1007/s00404-026-08389-0

**Published:** 2026-04-10

**Authors:** Werner Rath, Panagiotis Tsikouras, Ulrich Pecks

**Affiliations:** 1 Medical Faculty, Obstetrics and Gynecology, University-Hospital Schleswig-Holstein, Campus Kiel, 24105 Kiel, Germany; 2 Department of Obstetrics, Hospital of Alexandroupolis, Democritus University of Thrace, Alexandroupolis, Greece; 3 Department of Obstetrics, Würzburg University Hospital, Josef-Schneider-Str. 4, 97080 Würzburg, Germany; 4 Institute of Midwifery Science, Würzburg University Hospital, Josef-Schneider-Str. 4, 97080 Würzburg, Germany

**Keywords:** Inherited thrombophilia, Venous thromboembolism, Prevention, Guidelines, Recommendations

## Abstract

Women with inherited thrombophilia represent more than 15% of the pregnant population. 20–50% of pregnancy-related venous thromboembolism (VTE) is associated with at least one inherited thrombophilia, which increases the risk of VTE up to 40-fold depending on the type of thrombophilia and the family history of VTE. Most societies consider homozygosity of factor V Leiden and the prothrombin gene mutation, compound heterozygosity of both, and severe deficiency of factor V Leiden and prothrombin gene mutation as “high risk” thrombophilia and heterozygous for factor V Leiden or the prothrombin gene mutation as “low risk” thrombophilia. Recommendations on pharmacological prophylaxis vary across international guidelines. According to expert consensus, pharmacological prophylaxis may be indicated if the risk threshold for VTE is > 3% balancing benefit against harm of heparin prophylaxis. In women with low-risk thrombophilia, guidelines recommend pharmacological prophylaxis ante- and postpartum only in cases with a positive family history of VTE or additional VTE risk factors. Most guidelines suggest heparin prophylaxis in women with homozygosity for factor V Leiden mutation or compound thrombophilia regardless of family history of VTE in the antenatal period and for 6 weeks after delivery. In women with protein S and protein C deficiencies, some guidelines advocate clinical surveillance rather than pharmacological prophylaxis antenatally, while postpartum pharmacological prophylaxis was recommended by all guidelines for women with a positive family history of VTE or with additional risk factors. Pharmacological prophylaxis in women with antithrombin deficiency remains a matter of debate and depends on the subtype and extent of antithrombin deficiency. There is yet no evidence from randomized, controlled trials that pharmacological prophylaxis significantly reduces the risk of VTE in women with inherited thrombophilia. Overestimation of the VTE risk and unnecessary use of heparin is an unsolved problem. Decision-making should also consider the increased risk of bleeding complications and wound hematoma associated with pharmacological thromboprophylaxis. Until there is more evidence for the benefit of pharmacological prophylaxis, the decision for or against prophylaxis remains a case-by-case decision taking into account the patient’s individual risk profile and woman’s preference.

## Introduction

Venous thromboembolism is a leading cause of maternal mortality accounting for 15% of maternal deaths in developed countries [1]. This results in approximately 1 per 100.000 pregnant women dying of pregnancy-related VTE [2, 3]. The absolute risk of VTE is 5 times higher in pregnancy and 15–35-fold higher in the early puerperium compared to the non-pregnant population of the same age group [4]. Of note, women with inherited thrombophilia represent more than 15% of the pregnant population and are at increased risk of VTE[5, 6]. Inherited thrombophilia is frequently diagnosed in women with VTE during pregnancy or the puerperium, with the heterozygous Factor V Leiden mutation accounting for 28%, the heterozygous prothrombin gene mutation or the combined heterozygous Factor V Leiden and heterozygous prothrombin gene mutation each accounting for 8%, and the homozygous Factor V Leiden mutation accounting for 2.6% [7].

Depending on the type of inherited thrombophilia, the risk of VTE in pregnancy and the puerperium is increased by 3–41-fold [8, 9] (Table 1). For instance, homozygosity for factor V Leiden is associated with a relative increase in VTE risk by a factor of 35, whereas heterozygosity of factor V Leiden has an eight-fold risk increase [8]. Given a baseline incidence of VTE of 0.1% in pregnant women, this results in an absolute risk of 3.4% and 0.8% [7, 10, 11]. The risk of VTE increases even further in the presence of additional risk factors. A personal or family history of VTE in women with PC or PS deficiency results in antepartum and postpartum risks of VTE of 1.7% (95% CI 0.4–8.9) and 6.6% (95% CI 2.2–14.7), respectively, while women with the same coagulation inhibitor deficiencies, but without a personal or family history VTE risk, are 0.7% (95% CI 0.3–1.5) and 0.5% (95% CI 0.2–1.0) [12].

**Table 1:** Risk of VTE in pregnant women with inherited thrombophilia

Type of inherited thrombophilia	Prevalence in general populations (%)	Absolute risk of VTE (%) of pregnancy (95% CI)	
		Non-family studies	Studies with positive family history
Heterozygous FVL	2.0–7.0	1.2 (0.8–1,8)	3.1 (2.1–4.6)
Heterozygous PGM	2.0	1.0 (0.3–2.6)	2.6 (0.9–5.6)
Homozygous FVL	0.2–0.5	4.8 (1.4–16.8)	14.0 (6.3–25.8)
Homozygous PGM	< 0.01	37 (0.2–78.3)	n.s
Protein C deficiency	0.2–0.3	0.7 (0.3–1.5)	1.7 (0.4–8.9)
Protein S deficiency	< 0.1–0.1	0.5 (0.2–1.0)	6.6 (2.2–14.7)
AT deficiency	< 0.1–0.6	0.7 (0.2–2.4)	3.0 (0.08–15.8)

Modified from Hart 2020 [12] and Middeldorp et al. 2022 [6]

*VTE* Venous thromboembolism,* FV Leiden* Factor V Leiden mutation, *PGM* prothrombin gene mutation,* AT* Antithrombin,* CI* Confidence interval

It is a matter of debate which type of thrombophilia should be classified as “high” or “low” risk [5, 13–15]. Most guidelines agree that heterozygous factor V Leiden and heterozygous prothrombin gene mutation are considered to moderate thrombophilia and homozygosity or compound heterozygosity for factor V Leiden, and prothrombin gene mutation is considered high-risk thrombophilia [12, 13, 15–19]. Protein S and C deficiencies are addressed in a significantly less uniform manner in the guidelines: only ACOG [15], AWMF [19], and GTH [12] consider these as high risk when activity is very low (PC activity < 60%, PS activity < 40%). AT deficiency is considered as high-risk thrombophilia by ACOG [15], RCOG [13], SOGC [18], GTH, and AWMF only in case of severe deficiency (AT < 60%) [12, 19].

Societies and guidelines therefore recommend pharmacological thromboprophylaxis for pregnant women at high risk of VTE in order to minimize the disadvantages for women's health and reduce the risk to the level of non-pregnant women. Yet, existing recommendations on thrombophilia in pregnant women with inherited thrombophilia are limited by a lack of evidence and rely mostly on case–control studies.

A major problem in decision-making is the uncertainty in setting clear thresholds for risk estimates in order to recommend prophylaxis and weigh the number of women to be treated to prevent an event against the number of women who are more likely to suffer harm from the intervention. Various guidelines recommend pharmacological prophylaxis only when the risk of thromboembolic events increases to 1 to 5% [5, 16–19]. A group of experts supports a risk threshold of 3% or more for pharmacological interventions and published this in a consensus statement [5]. However, according to a systematic review and Bayesian meta-analysis of 36 studies, this risk threshold of 3% is only exceeded in women with AT, PC, or PS deficiency, or homozygosity for factor V Leiden. Of note, all women with AT, PS, and PC deficiencies included in this meta-analysis had a positive family history of VTE [20].

Given the wide range of different recommendations and the uncertainty among physicians when recommending pharmacological thromboprophylaxis, this review aimed to summarize the various guidelines on the prevention of VTE in pregnant women with hereditary thrombophilia.

### Recommendations from current guidelines

1. Heterozygosity for factor V Leiden or prothrombin gene mutation (Table 2)

**Table 2:** Prevention of first VTE in pregnant women with hereditary thrombophilia (in extracts)

Heterozygosity for Facor V Leiden or prothrombin gene mutation				
	ACCP 2012	SOGC 2014	RCOG 2015	ACOG 2018
Antepartum	Clinical surveillance regardless of family history of VTE (2C)	Clinical surveillance (no grade)	Clinical surveillance unless additional risk factors score > 3 LMWH prophylaxis throughout pregnancy score 2 from 28 weeks (Grade D)	Either clinical surveillance or prophylactic LMWH (no grade)
Postpartum	Clinical surveillance positive family history of VTE LMWH prophylaxis for 6 weeks (2 C)	Clinical surveillance combination with only 2 risk factors (absolute risk of each < 1%) LMWH prophylaxis for 6 weeks (no grade)	LMWH prophylaxis for at least 10 days in case of additional risk factors (score > 1) family history of VTE in a first-degree relative 6 weeks (Grade D)	Either clinical surveillance or heparin prophylaxis if there are additional risk factors (e.g., first-degree relatives with VTE < 50 years)

*LMWH* low molecular weight heparin, *ACCP* American college of chest physicians, *SOGC* Society of obstetrics and gynaecology of Canada, *RCOG* Royal college of obstetricians and gynaecologists, *ACOG* American college of obstetricians and gynecologists

*Antepartum*: The ACCP [16] suggested only antepartum clinical surveillance, regardless of family history of VTE (Grade C), which is in accordance with the SOGC guidelines [18] and the ASH guidelines [17].

The RCOG also recommended clinical surveillance unless additional risk factors are present. In case of a weighted score of > 3, LMWH prophylaxis should be given throughout pregnancy, and if the weighted score is only 2, pharmacological prophylaxis should be considered from 28 weeks (Grade D) [13]. The ACOG suggested surveillance in women without previous VTE and with a family history (first-degree relatives) of VTE. However, in the latter women, heparin prophylaxis may also be considered, and a prophylactic or intermediate dose of LMWH/UFH in women with a single previous episode of VTE—not receiving long-term anticoagulation therapy [15]. The GTH came to similar recommendations, advocating no pharmacological thromboprophylaxis if there are no prior VTE and no additional VTE risk factors [12].

*Postpartum*: The ACCP guidelines suggested clinical surveillance and LMWH prophylaxis in women with a positive family history of VTE or additional risk factors [16] which is in line with the ACOG guidelines [15] and the SOGC guideline, which proposed pharmacological prophylaxis in combination with 2 other risk factors (each with an absolute risk of VTE < 1% in isolation) for 6 weeks postpartum [18].

According to the RCOG guidelines [13], thromboprophylaxis should be considered for at least 10 days after delivery if additional risk factors are present with a weighted score of > 1.

In women with a family history of VTE (first-degree relatives), LMWH prophylaxis should be administered for 6 weeks postpartum (Grade D).

On the other hand, the ASH guidelines suggested against pharmacological prophylaxis regardless of family history of VTE [17].

2. Homozygosity for factor V Leiden or prothrombin gene mutation (Table 3)

**Table 3:** Prevention of the first VTE in pregnant women with hereditary thrombophilia (in extracts)

	Homozygosity Factor V Leiden or Prothrombin gene mutation			
	ACCP 2012	SOGC 2014	RCOG 2015	ACOG 2018
Antepartum	Clinical surveillance, if there is no family history of VTE LMWH prophylaxis (prophylactic or intermediate dose) in the presence of a positive family history of VTE (Grade 2B)	Prophylactic LMWH for FVL (II 2 A) and for PGM (III B)	LMWH prophylaxis should be considered (Grade D)	Prophylactic LMWH (no grade)
Postpartum	LMWH prophylaxis for 6 weeks regardless of family history of VTE (Grade 2B)	Prophylactic LMWH for 6 weeks (II 3B)	LMWH prophylaxis for 6 weeks postpartum (Grade D)	Postpartum anticoagulation (no grade)

*LMWH* Low molecular weight heparin,* FV Leiden* Factor V Leiden mutation,* PDM* Prothrombin G20210A gene mutation, *ACCP* American College of Chest Physicians, *SOGC* Society of Obstetrics and Gynaecology of Canada, *RCOG* Royal College of Obstetricians and Gynaecologists, *ACOG* American College of Obstetricians and Gynecologists

*Antepartum*: The SOGC (Grade II 2 A, IIIB), RCOG (Grade D), and ACOG (no grade) guidelines recommended prophylactic LMWH/UFH prophylaxis [13, 15, 18], the ACCP guidelines [16], only in the presence of a positive family history of VTE (Grade 2B) and clinical surveillance in women with no family history of VTE (Grade 2B).

The ASH guidelines suggested pharmacological prophylaxis for women who were homozygous for the factor V Leiden mutation regardless of family history and no heparin prophylaxis for women homozygous for the prothrombin gene mutation without a family history of VTE.

Despite the lack of evidence, panel members favored prophylaxis in these women with a family history of VTE [17]

*Postpartum*: All guidelines recommended LMWH thromboprophylaxis mostly for 6 weeks postpartum in a prophylactic or intermediate dose.

3. Compound heterozygosity for factor V Leiden and prothrombin gene mutation (Table 4)

**Table 4:** Prevention of first VTE in pregnant women with hereditary thrombophilia (in extracts)

Compound heterozygosity for Factor V Leiden and Prothrombin gene mutation				
	ACCP 2012	SOGC 2014	RCOG 2015	ACOG 2018
Antepartum	Clinical surveillance regardless of family history of VTE (Grade 2 C)	Prophylactic LMWH (III B)	LMWH prophylaxis should be considered (Grade D)	Prophylactic LMWH (no grade)
Postpartum	Clinical surveillance, if no family history of VTE LMWH prophylaxis (prophylactic or intermediate dose) for 6 weeks, if there is a family history of VTE (Grade 2B)	LMWH prophylaxis for 6 weeks (II 3B)	LMWH prophylaxis for 6 weeks postpartum (Grade D)	Postpartum anticoagulation (no grade)

*LMWH* Low molecular weight heparin

*Antepartum*: There is general agreement among guidelines that these women need prophylactic LMWH throughout pregnancy regardless of family history except the ACCP guidelines [16] proposed clinical surveillance regardless of family history of VTE (Grade C).

*Postpartum*: Prophylactic LMWH prophylaxis is recommended by all guidelines for 6 weeks postpartum regardless of family history of VTE; only the ACCP guidelines suggested against pharmacological prophylaxis if there is no family history. In these cases, clinical surveillance may be sufficient (Grade 2 C).

4. Protein C (PC) and protein S (PS) deficiency (Table 5)

**Table 5:** Prevention of first VTE in pregnant women with hereditary thrombophilia (in extracts)

Protein C and Protein S deficiency				
	ACCP 2012	SOGC 2014	RCOG 2015	ACOG 2018
Antepartum	Clinical surveillance regardless of family history of VTE (Grade 2 C)	Clinical surveillance (no grade)	LMWH prophylaxis should be considered (Grade D)	Either clinical surveillance or prophylaxis LMWH (no grade)
Postpartum	Clinical surveillance, if no family history of VTE LMWH prophylaxis for 6 weeks, if there is a family history of VTE (Grade 2 C)	Clinical surveillance or prophylaxis if present in combination with only 2 risk factors (each with an absolute risk < 1%) for 6 weeks (II 3B)	LMWH prophylaxis for 6 weeks postpartum (Grade D)	Either clinical surveillance or anticoagulation if there are additional risk factors (e.g., first-degree relatives with VTE < 50 years) (no grade)

*LMWH* Low molecular weight heparin

*Antepartum*: While the SOGC, ACCP, and ASH guidelines suggested clinical surveillance without pharmacological thromboprophylaxis [16–18], the ACOG guidelines considered heparin prophylaxis in women with a family history of VTE (first-degree relatives) and in women with a single previous episode of VTE [15] RCOG recommended LMWH prophylaxis independent of personal or family history of VTE (Grade B). The GTH came to similar recommendations and pointed out that patients with severe reduction of activity levels, a positive family history, or additional risk factors should receive pharmacological prophylaxis throughout pregnancy and for 6 weeks after delivery [12].

*Postpartum*: The ASH guidelines suggested pharmacological prophylaxis only in women with a family history of VTE [17], and the RCOG recommended LMWH prophylaxis for 6 weeks postpartum (Grade D; 13). The SOGC [18] proposed clinical surveillance or pharmacological prophylaxis if there are more than 2 additional risk factors every with an absolute risk of VTE < 1% in isolation (risk factors are specified in the guidelines). The ACOG advocated clinical surveillance and prophylactic heparin prophylaxis if the patient has additional risk factors, namely a family history of VTE or a single previous episode of VTE [15], and the ACCP [16] suggested pharmacological prophylaxis for 6 weeks postpartum only in women with a family history of VTE (Grade 2 C).

5. Antithrombin deficiency (Table 6)

**Table 6:** Prevention of first VTE in pregnant women with hereditary thrombophilia (in extracts)

Antithrombin deficiency				
	ACCP 2012	SOGC 2014	RCOG 2015	ACOG 2018
Antepartum	Clinical surveillance regardless of family history of VTE (Grade 2 C)	Prophylactic LMWH (III B)	LMWH prophylaxis from at least 28 weeks additional risk factors (score > 1) prophylaxis from the first trimester (Grade D)	Prophylactic LMWH (no grade)
Postpartum	Clinical surveillance, if no family history of VTE LMWH prophylaxis (prophylactic or intermediate dose) for 6 weeks, if there is a family history of VTE (Grade 2 C)	Prophylactic LMWH for 6 weeks (II 2B)	LMWH prophylaxis for at least 6 weeks (Grade D)	Postpartum anticoagulation (no grade)

*LMWH* low molecular weight heparin

*Antepartum*: While the ACCP suggested clinical surveillance regardless of family history of VTE (Grade 2 C; 16), the ASH recommended pharmacological prophylaxis only in patients with a family history of VTE [17]. Other guidelines [13, 15, 18] consistently favored the prophylactic use of LMWH/UFH in these women. With respect to the RCOG guidelines, women without additional risk factors should be provided with LMWH prophylaxis from at least 28 weeks. If additional risk factors with a weighted score of at least 1 are present, the prophylaxis should be provided from the first trimester (Grade D; 13).

*Postpartum*: The SOGC (Grade 2B), RCOG (Grade D), and ACOG (no Grade) guidelines favored LMWH prophylaxis given for 6 weeks after delivery [13, 15, 18] while the ASH and ACCP (Grade 2 C) guidelines suggested against pharmacological prophylaxis for women without a family history of VTE, but recommended prophylaxis for women with a family history of VTE (16, 17)

Table 7 summarizes the recommendations of the American Society of Hematology 2018.

**Table 7:**
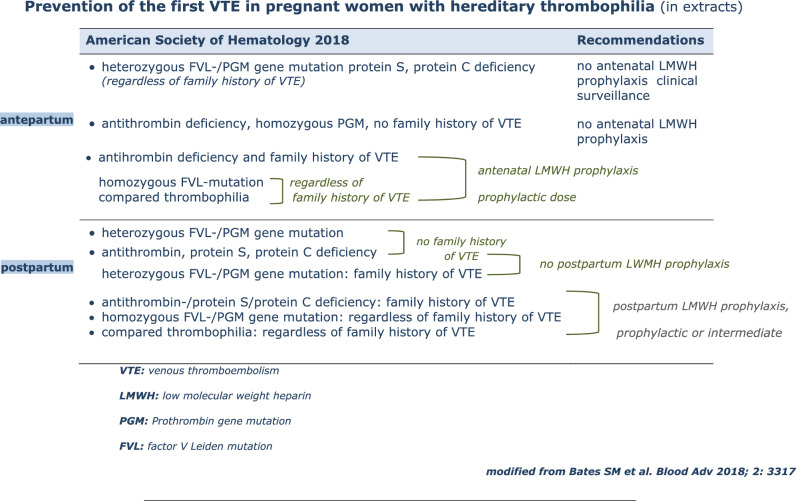
Prevention of the first VTE in pregnant women with hereditary thrombophilia (in extracts)

*VTE* venous thromboembolism,* LMWH* low molecular weight heparin,* PGM* Prothrombin gene mutation,* FVL* factor V Leiden mutation, modified from Bates SM et al. Blood Adv 2018; 2: 3317

## Discussion

Pregnancy increases the risk of developing VTE in women. Women with hereditary thrombophilia are mostly at risk, and often hereditary thrombophilia is only diagnosed after VTE has occurred during pregnancy [6].

There is limited data on the degree of thrombotic risk associated with inherited thrombophilia and on the interaction of risk factors which makes it difficult to develop evidence-based risk stratification for managing thrombosis prophylaxis.

Hence, there is a lack of consensus on thromboprophylaxis in international guidelines [13, 15, 17–19]. Inconsistencies in guideline recommendations may result from the use of different risk thresholds for suggesting prophylaxis [5].

A further limitation may be that the risk estimates are often imprecise, particularly for less common thrombophilia [5].

However, the assessment of the patient’s individual risk during pregnancy and puerperium is mandatory to provide evidence-based thromboprophylaxis management.

The decision to use anticoagulation in women with thrombophilia is influenced, particularly, by personal and family history of VTE, severity of thrombophilia, and additional VTE risk factors [15].

Hereditary thrombophilia can roughly be categorized in common with moderate VTE risk (heterozygous factor V Leiden and heterozygous prothrombin gene mutation) and rare with high risk (homozygous for factor V Leiden and the prothrombin gene mutation, severe deficiency of PS, PC, or AT), although definitions of low- versus high-risk thrombophilia vary across guidelines [12].

In women with moderate VTE risk, guidelines did not generally recommend pharmacological prophylaxis in the antenatal and postpartum period. In women with a positive family history of VTE or with additional VTE risk factors, guidelines suggested prophylactic LMWH [12, 13, 15–18], while the ASH guidelines favored no VTE prophylaxes in pregnant women with a positive family history of VTE [17].

According to current guidelines, homozygosity for factor V Leiden and prothrombin gene mutation are indicators for antenatal pharmacological prophylaxis, which should be continued for 6 weeks postpartum [13, 15, 16, 18]. The ASH guidelines suggest against pharmacological prophylaxis in pregnant women with homozygosity for the prothrombin gene mutation regardless of the family history of VTE [17].

All guidelines except the ACCP guideline [16] recommended prophylactic LMWH in pregnant women with combined thrombophilia in pregnancy and for 6 weeks postpartum [12, 13, 15, 18] regardless of family history of VTE [17].

These are some inconsistencies between guidelines regarding recommendations of pharmacological prophylaxis in women with coagulation inhibitor deficiencies. While some guidelines [16–18] advocated only clinical surveillance rather than pharmacological prophylaxis in pregnant women with protein S or protein C deficiency, the RCOG guidelines [13] stated that LMWH prophylaxis should be considered, and the GTH suggested pharmacological prophylaxis in case of severe reduction of activity levels for PS or PC [12].

Considering postpartum prophylaxis, clinical surveillance was consistently recommended and pharmacological prophylaxis for 6 weeks after delivery for women with a family history of VTE and/or additional VTE risk factors [12, 13, 15, 17, 18].

Patients with AT deficiency are supposed to be at a higher risk of VTE than patients with PS or PC deficiency; however, the absolute risk estimates are uncertain and vary widely depending on the subtype and extent of the AT deficiency [6], which is reflected in the different guideline recommendations. Three guidelines suggested antenatal prophylactic LMWH [13, 15, 18], one guideline only for women who have a family history of VTE [17], and further guidelines, only clinical surveillance regardless of family history of VTE [16].

Postpartum pharmacological prophylaxis for 6 weeks was favored by 3 guidelines [13, 15, 18], while three guidelines recommended LMWH prophylaxis only in women with a positive family history of VTE [16, 17], or with severe AT deficiency or additional VTE risk factors [12].

Notably, all recommendations of current guidelines regarding pharmacological VTE prophylaxis in women with inherited thrombophilia are of low certainty in evidence [6].

Some guidelines may overestimate the risk of clinically significant VTE in hospitalized women because they relied on epidemiological studies with wide ranges in VTE risk factors. This may lead to an unnecessary use of LMWH associated with higher rates of bleeding complications and incremental cost [21, 22].

It is not surprising that guidelines differ in recommendations for pharmacological VTE prophylaxis [23–25]. For example, in a recent cross-outcome study evaluating the rate of postpartum LMWH prophylaxis applied by the RCOG guidelines, 53.6% of women would have qualified for postpartum LMWH compared to 8.3% using ACCP guidelines [26].

There is low evidence from randomized, controlled trials (RCT) that ante- or postpartum pharmacological prophylaxis in women with inherited thrombophilia reduces the risk of VTE significantly. Such studies seem to be not feasible since a tremendously high number of patients are needed to demonstrate a clear effect of pharmacological prophylaxis compared to no treatment. Considering the significantly increased risk of VTE in high-risk thrombophilia, it may be a matter of debate if placebo-controlled studies or studies without prophylaxis are ethically justified.

On the other hand, benefits and harms of pharmacological prophylaxis must be balanced against each other in an individualized manner.

This risk of major bleeding has been estimated at 0.4–4%, and the relative risk of severe bleeding has been calculated at 1.48 (95% CI 0.25–8.72) [27, 28].

In addition, the rate of wound hematoma attributable to LMWH prophylaxis has been reported to be approximately 2% compared to women without prophylaxis (0.4%) [29].

The cost-effectiveness of various thromboprophylaxis strategies with LMWH has been examined [30]. Pharmacological VTE prophylaxis with LMWH in pregnancy is cost-effective for high-risk patients such as those with prior VTE or high-risk thrombophilia [22], while no prophylaxis appears to be less costly than prophylaxis in low-risk pregnant women when balancing prevention benefits against bleeding harms [31].

Current risk assessment models have weak predictive accuracy for VTE and their uses are not recommended in daily practice for decision-making [32]. The obstetricians’ decision may also be influenced by the fear of medico-legal conflict in the event of pulmonary embolism if they miss significant VTE risk factors and withhold pharmacological VTE prophylaxis.

Given the lack of evidence from randomized, controlled trials, the decision for or against pharmacological prophylaxis in pregnant women with thrombophilia remains a case-by-case decision taken into account the patient’s individual risk profile and the woman’s preference after comprehensive patient counseling, not neglecting potential LMWH-related side effects.

## Data Availability

No datasets were generated or analyzed during the current study.

## Abbreviations

VTE: Venous thromboembolism
p.c.: Protein C
p.s.: Protein S
fVLM: Factor V Leiden mutation
PGM: Prothrombin gene mutation
AT: Antithrombin
ACOG: American College of Obstetricians and Gynecologists
RCOG: Royal College of Obstetricians and Gynaecologists
ACCP: American College of Chest Physicians
ASH: American Society of Hematology
GTH: German Society of Thrombosis and Hemostasis
AWMF: Arbeitsgemeinschaft der Wissenschaftlichen Medizinischen Fachgesellschaften
S_3_ Leitlinie: Prophylaxe venöser Thromboembolien (VTE)
SOGC: Society of Obstetricians and Gynecologists of Canada
LMWH: Low-molecular-weight heparin
UFH: Unfractionated heparin
